# Orientirung

**Published:** 1911-06-17

**Authors:** 


### "ORIENTIRUNG."

By A MEDICAL SUPERINTENDENT.

It is as evident a proposition as can be, that if ever the medical profession had need to take its bearings for a course of united action, it is now. The Government of the country, speaking, by no very extraordinary chance, by the mouth of a lawyer, has made proposals for a scheme of medical benefit for the bulk of the nation; a scheme which everyone must acknowledge to be necessary, but which on the face of it involves the thinking-out of thousands and thousands of details, neglect of persevering attention to which may easily wreck the whole purpose, to the grief and discomfiture of all friends of rightful progress. The stir in our own ranks is such as has not been seen for a generation, and there are signs that it has been to some purpose. Mr. Lloyd George's recent heckling reflected, on the whole, credit to all parties. That accomplished persuader and bargainer exerted his undeniable powers fully. Where the Chancellor of the Exchequer yielded, he yielded gracefully, like a sensible man; and like a good negotiator, too, when he stood out, he made considerable show of his best weapon, the bâton of official authority. Details vitally important to medical men will, it is freely conceded, be settled by an *ad hoc* body, namely, the Insurance Commissioners guided by an Advisory Committee. But the prospective medical members of the latter are warned only to tender advice and not to attempt advocacy of the interests of their profession. One can easily perceive that, so to say, what is a warning in the green stick may become a pretty effective regulation in the dry one. Further, the medical men present were told at an early stage of the proceedings, before most of the pledges had been given, that the Bill would be passed in a couple of months' time. Well, it may be, and every conscientious medical man who has seen the misery of the masses, and therefore shares a famous economist's opinion that something like a revolution in their condition is desirable, will hope so, provided that it is properly amended in this short interval. But we give political leaders credit for enough common sense—a quality they do not lack—not to try to thrust on doctors anything in this kind whch they show themselves unwilling to take. At the worst and lowest, there are nothing like enough of the dregs and unfortunates of the profession to half-man such a service as is contemplated. It takes six years to make a doctor, but a good deal less time, on occasion, to see out a Ministry. And the fear of committing an illegal act will hardly stop a man with a bone in his throat from sending for the doctor.

All this may sound like self-interest, which is a cry easily raised and not so easily met. But what has not yet been sufficiently pointed out is that the medical profession is no aggregation of prosperous, acquisitive sybarites. Its members are hard-working, essentially middle-class, unused therefore to luxury, privilege or indulgence, thoughtless and unbusinesslike over money matters, as much on the stretch physically and mentally as any successful business man, but earning a small fraction of his income; and supposed to be rewarded by a cheap sentimental halo of publicism, of philanthropy, and perhaps of scientific culture. Their ideas then as to pecuniary remuneration will not be on the commercial or aristocratic scale, will not be extravagant. We wonder, too, what rate of overtime a big trades union would approve of for anxious nightwork three times every week, which is, of course, the lot of most of those Sir Thomas Barlow calls, rather mouthingly, " our humbler brethren."

An article on the present subject might run to any length, and in scores of different directions, but there is a point which seems to come in here, which is that to be of service, our *orientirung* must be organised and co-ordinated. As medical champions, reckoning medical corporations alone, there have been three or four Richmonds in the field already. No doubt these will in proper time agree upon an expression of opinion, and by so doing give real strength to the hands of reformers, and incidentally establish a valuable precedent. For if there be a profession which, in gross and large, should be trusted to act in accordance with the spirit of the time, it is medicine. Theology is decaying and law much where it was in the days of the last great empire, but the renewals of progress in medicine and in democracy have been two chief events in human history of the last thirty years. Yet, whereas votaries of the Church and the Law exert real direct governing influence, medicine has none. Not that it is without faults especially dangerous to the great scheme now foreshadowed, faults which, to come to particulars, Mr. Lloyd George was no doubt too tactful to indicate plainly as such. " Ruthlessly checked " is plain speaking, but just the right words to use in connection with malingering. In the measure of the possible, what are technically known as " placebos " and " conversation calls " are things to be struck at vigorously. It is sad to think of the time and money now wasted in giving drug decoctions to hypochondriacs and other unprofitable members of society. But the great point now is that, the Bill having been read, there should be time for this mutual criticism before anybody is committed to anything. All should take plenty of thought before the great tower is begun.

